# NOTCH Signaling in Aortic Valve Development and Calcific Aortic Valve Disease

**DOI:** 10.3389/fcvm.2021.682298

**Published:** 2021-06-22

**Authors:** Yidong Wang, Yuan Fang, Pengfei Lu, Bingruo Wu, Bin Zhou

**Affiliations:** ^1^The Institute of Cardiovascular Sciences, School of Basic Medical Sciences, Xi'an Jiaotong University Health Science Center, Xi'an, China; ^2^Department of Genetics, Albert Einstein College of Medicine, Bronx, NY, United States; ^3^Departments of Genetics, Pediatrics (Pediatric Genetic Medicine), and Medicine (Cardiology), The Wilf Family Cardiovascular Research Institute, Albert Einstein College of Medicine, Bronx, NY, United States; ^4^The Einstein Institute for Aging Research, Albert Einstein College of Medicine, Bronx, NY, United States

**Keywords:** NOTCH, aortic valve development, calcific aortic valve disease, EMT, bicuspid aortic valve

## Abstract

NOTCH intercellular signaling mediates the communications between adjacent cells involved in multiple biological processes essential for tissue morphogenesis and homeostasis. The *NOTCH1* mutations are the first identified human genetic variants that cause congenital bicuspid aortic valve (BAV) and calcific aortic valve disease (CAVD). Genetic variants affecting other genes in the NOTCH signaling pathway may also contribute to the development of BAV and the pathogenesis of CAVD. While CAVD occurs commonly in the elderly population with tri-leaflet aortic valve, patients with BAV have a high risk of developing CAVD at a young age. This observation indicates an important role of NOTCH signaling in the postnatal homeostasis of the aortic valve, in addition to its prenatal functions during aortic valve development. Over the last decade, animal studies, especially with the mouse models, have revealed detailed information in the developmental etiology of congenital aortic valve defects. In this review, we will discuss the molecular and cellular aspects of aortic valve development and examine the embryonic pathogenesis of BAV. We will focus our discussions on the NOTCH signaling during the endocardial-to-mesenchymal transformation (EMT) and the post-EMT remodeling of the aortic valve. We will further examine the involvement of the NOTCH mutations in the postnatal development of CAVD. We will emphasize the deleterious impact of the embryonic valve defects on the homeostatic mechanisms of the adult aortic valve for the purpose of identifying the potential therapeutic targets for disease intervention.

## Introduction

The aortic valve ensures the unidirectional flow through the heart to deliver the oxygenated blood and nutrients to the body through cyclic pumping (1, 2). Over an average lifetime, the heart valves open and close over a billion times (2). The constant mechanical stress causes wear and tear of the heart valves that may lead to calcific aortic valve disease (CAVD) when the valve homeostatic mechanism is exhausted by repeated damages (3, 4). CAVD is the third leading cause of heart disease, and its incidence increases with age (5–12). Aortic valve replacement remains the only effective treatment option available for CAVD, as currently no medicine is able to prevent the disease progression (8, 9).

More than half of the patients with severe CAVD who need aortic valve replacement have a bicuspid aortic valve (BAV), a congenital defect affecting ~2% of the general population (9, 13, 14). These patients develop CAVD decades earlier than those who have a tri-leaflet aortic valve (1). These observations support that BAV-asociated CAVD has a developmental origin, and its postnatal stenotic progression by calcification is triggered by external stimuli as a second hit after the primary structural defect *in utero* (9). In support of this view of the pathogenesis of CAVD, the developmental defect of the aortic valve can cause abnormal flow dynamics and thereby predispose the valve to endothelial dysfunction and inflammatory activation, leading to earlier calcification in adulthood (9, 15–18). Therefore, better understanding of molecular mechanisms regulating aortic valve development, as well as postnatal aortic valve homeostasis, is required for the development of new therapeutic strategies for CAVD.

During embryogenesis, aortic valve leaflets are made up of valve endocardial cells (VECs) and valve interstitial cells (VICs), with VICs arising from VECs, cardiac neural crest cells (NCCs), or the second heart field (SHF) cardiomyocytes (19–25). Aortic valve development consists of multiple morphological and cellular events that are spatiotemporally coordinated by complex molecular signal networks (1). In the past two decades, researchers have made significant advance in understanding these developmental events and underlying molecular signaling mechanisms by using the experimental approaches including human genetics, animal models, and *in vitro* cell systems. Among the important discoveries, the NOTCH signaling is recognized as an essential molecular regulator of aortic valve development, which plays critical roles at multiple phases of valvulogenesis (26–31). Furthermore, the *NOTCH1* mutations are the first genetic variants identified for congenital BAV and acquired CAVD in human (32). Therefore, we will focus our review on the findings of the roles of NOTCH signaling in aortic valve development and embryonic pathogenesis of BAV, as well as their implications in the postnatal valve homeostasis and CAVD.

## Morphogenesis Of The Mammalian Heart Valve

The mammalian heart has four valves including atrioventricular (AV) valves and semilunar valves. The AV valves consist of mitral and tricuspid valve, which connects the left or right atrium to the left or right ventricle, and control the blood flow from the atria entering the ventricles (1). The semilunar valves include the aortic and pulmonary valve, which direct the blood flow from the left or right ventricle into the systemic or pulmonary circulation through the aorta or pulmonary artery (1). In all the heart valves, the mature valve leaflets are made up of the surface VECs and the core VICs, which are embedded in the well-organized extracellular matrix (ECM) (4, 8).

**Figure 1:**
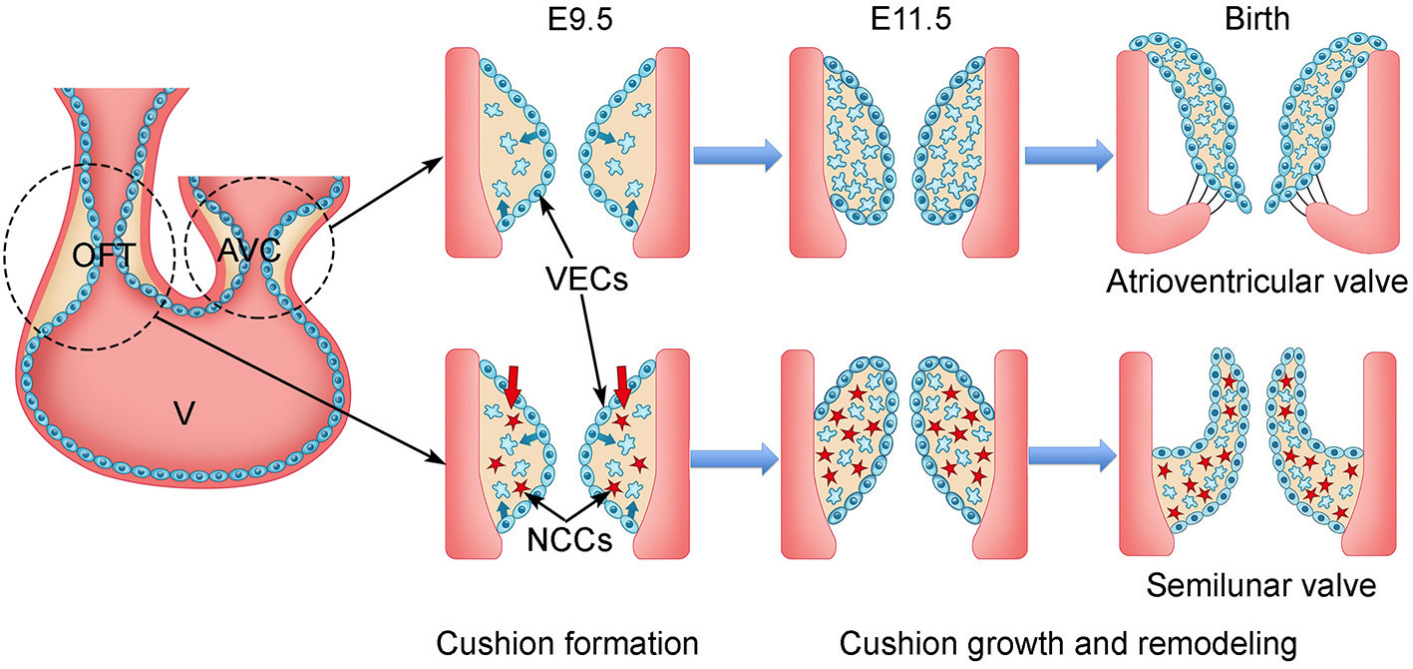
Schematic of cardiac valve development in mice. During mouse embryogenesis, the linear heart tube, composed of outer layer of myocardium (orange) and inner layer of endocardium (blue), undergoes looping beginning at E8.0. The process divides the heart tube into four segments: the atrium, atrioventricular canal (AVC), ventricle (v), and outflow tract (OFT) by E9.5. Two matrix-rich and acellular tissue swellings, named endocardial cushions, are formed within the AVC and OFT regions. In response to the signals from the myocardium, the endocardial cells within the AVC and proximal OFT regions undergo endocardial-to-mesenchymal transformation (EMT). The transformed mesenchymal cells invade the matrix-rich cardiac jelly and proliferate to populate the endocardial cushions within the proximal OFT, while the cardiac neural crest cells (NCCs) migrate into the distal OFT. By E11.5, the AVC and OFT cushions are fully cellularized. After E11.5, the AVC and OFT cushions grow rapidly and undergo intensive remodeling involving spatiotemporal cell proliferation and apoptosis of valve interstitial cells (VICs), as well as dynamic extracellular matrix (ECM) arrangement. The AVC and OFT cushions eventually give rise to the atrioventricular valves (mitral and tricuspid valve) and semilunar valves (aortic and pulmonary valve), respectively. VECs, valve endocardial cells.

During mouse embryogenesis and at embryonic day 7.5 (E7.5), a subset of mesodermal cells located at the anterior part of the primitive streak migrates to the splanchnic mesoderm and forms the cardiac crescent consisting of the first heart field (FHF) and SHF (23–25, 33, 34). At E8.5, the FHF-derived progenitors fuse at the midline of the embryo and become the primitive heart tube, which comprises an outer layer myocardium, an inner layer endocardium, and a sandwiched ECM-rich space, named cardiac jelly (35, 36). The primitive heart tube elongates by adding cells from the SHF to its both ends while simultaneously undergoing rightward looping to form an S-shape primitive heart consisting of the common atrium, AV canal (AVC), common ventricle, and outflow tract (OFT) (37–40). Around E9.5, heart valve development starts with the formation of endocardial cushions within the AVC and OFT regions through endocardial-to-mesenchymal transformation (EMT) (25, 41–43) (Figure 1). EMT is regulated by molecular signals from both myocardium and endocardium. In response to the bone morphogenic protein (BMP)/transforming growth factor β (TGF-β) signals from the myocardium, a subset of VECs begins to lose their surface markers of endothelial cells and gain the characteristics of mesenchymal cells (44–47). The transformed VECs then migrate into the cardiac jelly and proliferate to form the VICs of endocardial cushions (47). The cellularized endocardial cushions function as valve primordia, which are subjected to complex remodeling through spatiotemporally regulated proliferation and apoptosis of VICs, as well as tightly controlled synthesis and organization of ECM (41, 42). The remodeling process continues until birth when the cellularized endocardial cushions eventually become the mature heart valves with thin leaflets.

Lineage tracing studies show that the majority of VICs in AV valves are from the endocardial precursor cells through EMT (20). In contrast, VICs in the semilunar valves are from three distinct populations including VECs, NCCs, and the SHF-derived cardiomyocytes (20). Regarding the aortic valve, genetic lineage tracing of NCCs and VECs using *Wnt1*^Cre^ and *Tie2*^Cre^, respectively, shows that VECs make the major contribution to the primordia of two coronary leaflets of the aortic valve, whereas NCCs have a minor contribution (20). In contrast, the SHF progenitors, known to contribute to the OFT myocardium and which are essential for the OFT development, have been recently shown to be the major cellular origin of the intercalated cushion mesenchyme, which gives rise to the non-coronary leaflet of the aortic valve (19, 21, 22, 48–51). Functional studies in animal models further confirm the lineage tracing results and link the signal dysregulations in these three progenitors to various congenital aortic valve defects, that is, different BAV subtypes (21, 22, 49, 50, 52).

## Molecular Signals Underlying Early Heart Valve Morphogenesis

The rightward looping of the primitive heart tube leads to the formation of morphologically distinct atria, AVC, ventricles, and the OFT (36). In addition, the myocardium of these regions is already molecularly and functionally different (47, 53). The myocardium at AVC and OFT provide the crucial molecular signals that induce VECs to undergo EMT (41, 45, 47). Biochemical and genetic studies in chick and mouse embryos have identified BMP/TGF-β signaling as a key inducer of EMT (54). Early studies using cultured chicken AVC tissues in a collagen gel assay have demonstrated that TGF-β2 and TGF-β3 are the main cytokines that induce VECs to undergo EMT (55, 56). However, studies in mice have found that only TGF-β2 is required for the initiation of EMT by the AVC explants using the similar collagen gel assay (56), and the *Tgfb2* or *Tgfb3* null mice have no obvious defects in the EMT process and endocardial cushions (57). In contrast, BMP2 and BMP4 are expressed exclusively by the AVC and OFT myocardium, respectively, during early valve development (58–61). Myocardial-specific disruption of *Bmp2* in mice blocks EMT and results in endocardial cushion defects at AVC, suggesting BMP2 is a crucial inducer of EMT (58). Molecular analysis of *Bmp2* knockout embryos by RNA *in situ* hybridization has revealed that loss of *Bmp2* leads to decreased expression of *Tgfb2*, indicating TGF-β2 acts downstream of BMP2 during EMT (58). BMP2 also promotes the expression of *Snai1* and its nuclear stability through inhibiting GSK3β (62). Furthermore, T-box transcriptional factor *Tbx2* is another downstream target of BMP2 and is expressed in the myocardium of AVC and OFT, but absent in the chamber myocardium (63). Molecular analysis has shown that the BMP2-TBX2 signaling axis is essential for specifying the AVC myocardium by repressing the chamber-myocardium gene program (54, 63). Consistently, loss of *Tbx2* in mice results in EMT and endocardial cushion defects at AVC and OFT, whereas overexpression of *Tbx2* induces ectopic expression of *Tgfb2* in the chamber myocardium and ectopic EMT by the chamber endocardial cells (63, 64). Together, the findings of these studies demonstrate local myocardial signals in AVC and OFT that dedicate the cell fate of VECs during EMT to cellularize the endocardial cushions as early valve primordia.

## The Notch Signaling Pathway

**Figure 2:**
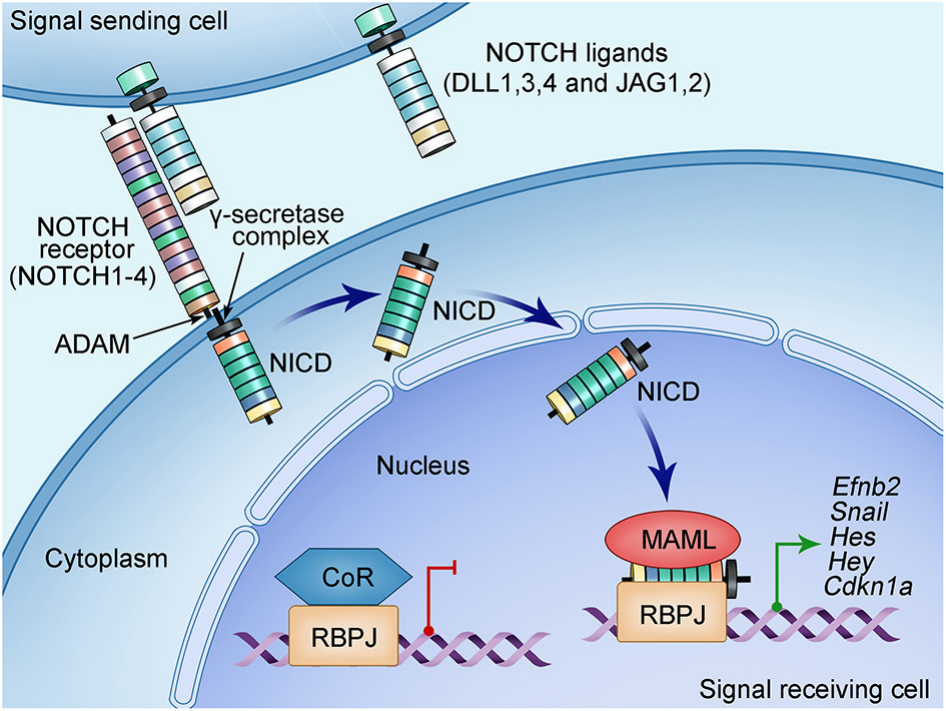
Schematic of NOTCH signaling pathway. NOTCH signaling pathway consists of ligands (DLL1, DLL3, DLL4, and JAG1, JAG2), receptor (NOTCH1-4), nuclear effector RBPJ, and cofactors. Both NOTCH ligands and receptors are type I transmembrane proteins with an extracellular domain consisting of a number of EGF-like repeats modified by sugars essential for the ligand-binding specificity. The NOTCH signal is initiated when the ligand on the surface of the signaling-sending cell binds the NOTCH receptor on the signaling-receiving cell. This ligand–receptor interaction generates a mechanical force that causes the conformational change of the NOTCH receptor, leading to subsequently proteolytic cleavages mediated by disintegrin and metalloproteinase domain-containing protein (ADAM) metalloproteinases and the γ-secretase–presenilin complex. These cleavages on the NOTCH receptor release the NOTCH intracellular domain (NICD), which then translocate into the nucleus and form a transcriptional complex with the DNA-binding protein RBPJ. RBPJ forms a complex with corepressors (CoR) and suppresses the transcription of target genes when NICD is not in the nucleus. The binding of NICD with RBPJ results in the release of CoR, while recruiting the coactivator Mastermind-like protein (MAML), leading to the transcription activation of the target genes such as the HES and HEY families of basic helix–loop–helix transcriptional repressors.

In addition to the aforementioned molecular signals from the AVC and OFT myocardium, others and we have studied the functions of NOTCH signaling in heart valve development. The NOTCH signaling pathway is highly conserved across species from zebrafishes to humans (26, 27, 65). It provides a major intercellular signaling mechanism that mediates the crosstalk between adjacent cells. The NOTCH pathway consists of the NOTCH ligands and receptors that are both single-pass transmembrane proteins at the cell surface (Figure 2). Mammals have four NOTCH receptors (NOTCH1-4) and five ligands that belong to two families: Serrate/Jagged families (JAG1,2) and Delta (DLL1,3,4) (26, 27, 65). A NOTCH receptor has a large extracellular fragment and a NOTCH intracellular domain (NICD) that contains two nuclear localization signals and a transactivation region. The pre-NOTCH proteins are processed in the Golgi apparatus through proteolytic cleavage mediated by a furin-like convertase. In addition, sugar residues are added to the epidermal growth factor–like repeats of the NOTCH extracellular domain by several glycosyltransferases including the Fringe family proteins and protein O-fucosyltransferases (29–31, 66–68). These posttranslational modifications determine the binding specificity between a NOTCH ligand and its receptor, thereby essential for the proper activation of the NOTCH signaling (69). The mature NOTCH proteins are then transferred to the cell surface and form a heterodimer receptor there through non-covalent interactions (66). The binding of the NOTCH ligand on a signal-sending cell to the NOTCH receptor on a signal-receiving cell leads to the endocytosis of the ligand, the process that generates mechanical force to pull on a NOTCH receptor. This pull action, in turn, triggers the conformational changes, leading to exposure of the S2 site of the receptor being recognized and further cleaved by the ADAM, a disintegrin and metalloproteinase (66). Further cleaving by γ-secretase at S3 site results in the release of the protein fragment NICD (70). The released NICD then shuttles into the nucleus and forms a complex with transcription factor RBPJ. Binding of NICD to RBPJ converts it from a repressor to an activator by releasing its corepressors, allowing recruitment of coactivator Mastermind (MAML) to activate gene expression. The well-known targeted genes include the basic–helix–loop–helix transcriptional factors of the *Hes* and *Hey* families (26, 27, 66).

The activation of the NOTCH signaling regulates multiple cellular functions including fate specification, differentiation, proliferation, apoptosis, and migration during embryogenesis and in adulthood (26). Studies using animal models have led to the identification of many critical roles of the NOTCH signaling in coordinating organ development and maintaining tissue homeostasis. In the cardiovascular system, the NOTCH signaling plays critical roles in early cardiomyocyte differentiation (71–75), ventricular trabeculation (76), cardiac valve formation (52, 59, 77, 78), OFT development (79), and angiogenesis (80), as well as cardiovascular homeostasis (26). The dysregulation of the NOTCH signaling is associated with congenital heart disease (32, 81–83) and several adult cardiovascular disease including atherosclerosis (84), hypertension (85–88), and CAVD (32, 89–91). Next, we will focus our discussion on the roles of the NOTCH signaling in aortic valve development and CAVD.

## Notch Signaling in Emt and Endocardial Cushion Formation

**Figure 3:**
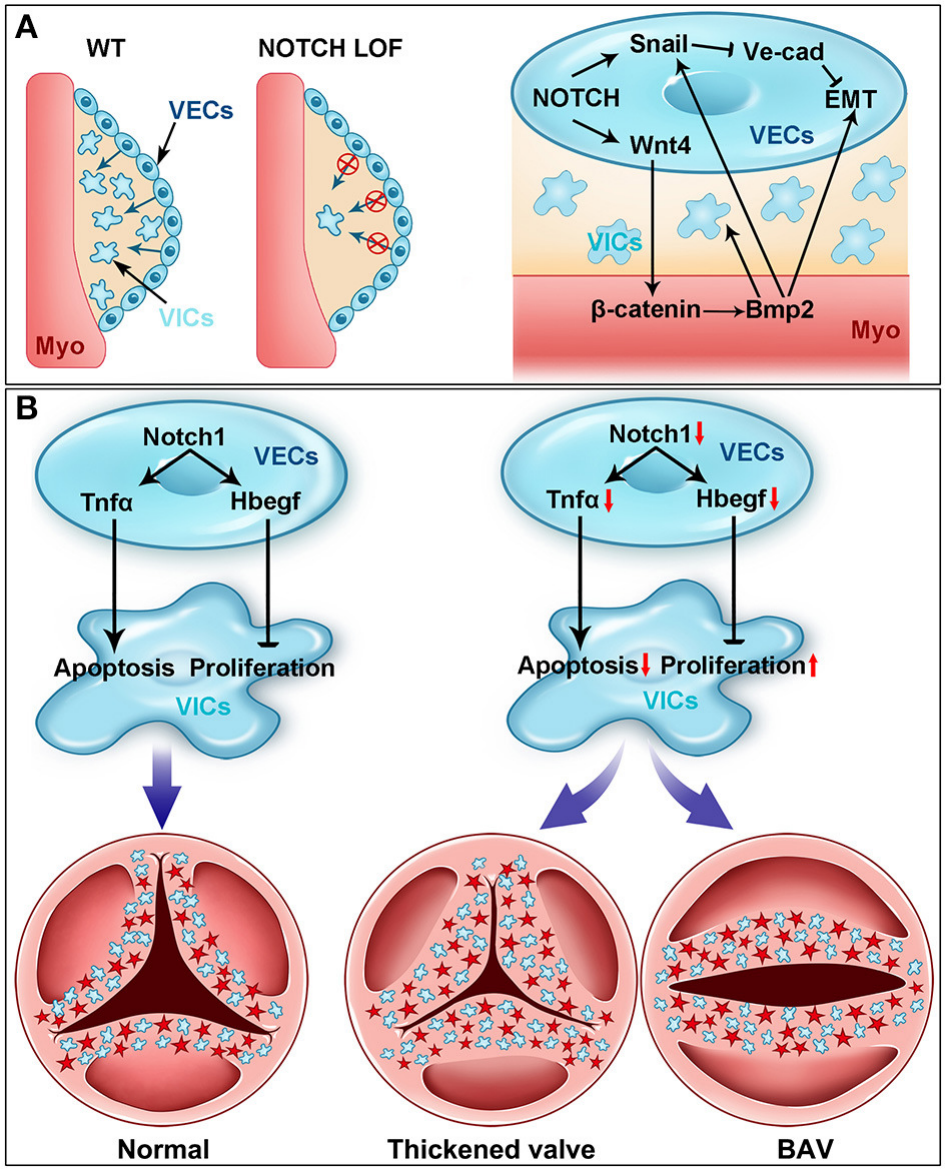
NOTCH signaling in cardiac valve development. **(A)** NOTCH is essential for early heart valve development (i.e., endocardial cushion formation). Cardiac valve development starts with the formation of endocardial cushions through the endocardial-to-mesenchymal transformation (EMT) (blue arrows) process in wild-type (WT) mouse embryos. The EMT process is severely blocked in the NOTCH loss-of-function (LOF) mutant embryos. Mechanistically, NOTCH activates the transcription of *Snail*, which subsequently represses the expression of *VE cadherin*. The downregulation of *VE cadherin* is prerequisite for the endocardial cells to delaminate the endocardial sheet and undergo EMT. NOTCH also induces the expression of *Wnt4* in the endocardium, which activates the canonical WNT signaling in the myocardium (Myo). The activation of WNT signaling in the myocardium promotes the expression of *Bmp2*, which feeds back to the endocardium through *Bmpr1* and regulates the EMT via stabilizing the *Snail* protein in the nucleus. *Bmp2* also promotes the proliferation of mesenchymal cells (MCs) during endocardial cushion formation. **(B)** NOTCH controls the cardiac valve remodeling through regulating the apoptosis and proliferation. NOTCH in the valve endocardial cells (VECs) promotes the expression of *Tnfa*, which subsequently induces the apoptosis of valve interstitial cells (VICs) originated from VECs (blue cells) and NCCs (red cells) during valvulogenesis. On the other hand, NOTCH in VECs directly activates the expression of *Hbegf*, which then inhibits the proliferation of VICs through repressing SMAD signaling. Genetic disruption of the NOTCH signaling pathway results in decreased expression of *Tnfa* and *Hbegf*, leading to the thickened valve leaflets and BAV, the most common congenital heart valve defect.

The NOTCH signaling in the endocardium is critical for the initiation of EMT and endocardial cushion formation (Figure 3A). Several NOTCH pathway genes (*Dll4, Jag1, Notch1, Notch4, Hey1*, and *Hey2*) are expressed in the endocardium within AVC and OFT regions (i.e., VECs) between E9.0 and E11.5 when EMT takes place in mice (59, 77, 78, 92, 93). Genetic disruption of *Notch1* or *Rbpj* severely blocks EMT and results in hypocellular endocardial cushions in mice (77). A similar phenotype was observed in mice with deletion of *Jag1* using either *Cdh5*^Cre^ or *Nfatc1*^Cre^ driver, suggesting *Jag1* is a key ligand inducing the NOTCH signaling during EMT and endocardial cushion formation (59, 93). In contrast, a recent study using *Tie2*^Cre^ driver demonstrated that *Dll4* but not *Jag1* is the key ligand in this process (78). This discrepancy might be due to different genetic backgrounds and/or *Cre* drivers used in these studies. In addition, the endothelial specific disruption of *Mib1*, an upstream regulator of the NOTCH signaling through modulating the ligand activity, recapitulated the EMT defect observed in the *Notch1* mutant mice (78). Consistent with these *in vivo* findings, the blockage of the NOTCH signaling by either genetic disruption of *Notch1* or *Rbpj* or treating wild-type AVC explants with the NOTCH inhibitor DAPT (*N*-[*N*-(3,5 difluorohenacetyl)-l-alanyl]-*S*-phenylglycine tert-butyl ester) severely impairs the EMT in the *in vitro* collagen gel assays (77).

Mechanistically, the NOTCH signaling directly regulates *Snai1* (*Snail*), which in turn represses the expression of VE cadherin, a major cell–cell adhesive protein expressed in the endothelial cells including VECs (77). Disruption of adhesion junction is prerequisite for VECs to delaminate the endocardial sheet at AVC and OFT, migrate, and invade the cardiac jelly. In addition, *in vitro* studies have demonstrated that *Snai2* (*Slug*) is a direct target of NOTCH1 and represses the expression of VE cadherin during EMT process (94). The EMT defect in the *Snai2* null AVC explants can be rescued by increased expression of *Snai1*, supporting a redundant function between *Snai1* and *Snai2* in regulation of EMT (94). Together, these findings have established a signaling axis consisting of NOTCH-SNAI1/SNAI2-VE cadherin that plays a key role in regulating EMT during endocardial cushion formation and early heart valve development. It is worth to mention that the JAG1/NOTCH signaling in the SHF derivatives also critically controls EMT in OFT through interaction with the FGF8-BMP4 signaling (95).

In addition to its VEC autonomous function, the NOTCH1 signaling in VECs also interacts with the signals from the myocardium to regulate EMT during endocardial cushion formation (59, 62). It is noted that while ventricular endocardial cells with ectopic activation of the NOTCH1 signaling acquires an EMT gene program, they are unable to migrate away from the cultured ventricular explants, but invasion can be triggered after additional BMP2 treatment (62). These observations suggest a synergic function between endocardial NOTCH and myocardial BMP signaling in promoting EMT. On the other hand, loss of myocardial *Bmp2* inhibits the expression of *Notch1* in VECs and EMT (58), whereas inhibition of the NOTCH signaling attenuates the invasive phenotype induced by BMP2 in cultured ventricular explants (62). To reveal the endocardial specific functions of the NOTCH1 signaling, we deleted *Notch1* in the endocardial cells of the developing mouse heart by using an *Nfatc1*^Cre^ driver (59). In contrast to the germline *Notch1* null mutants, which have severe overall vascular defects that affect early cardiac morphogenesis (76, 80), our *Notch1* conditional knockout embryos develop normal vascular networks, thereby allowing us to specifically dissect the endocardial-specific roles of NOTCH1 signaling in cardiac development (59). Our studies confirmed that deletion of *Notch1* in the endocardial cells impairs EMT and results in hypocellular endocardial cushions. Furthermore, we show that endocardial NOTCH1 is required for maintaining the myocardial *Bmp2* expression through WNT4-mediated paracrine regulation (59). Our studies demonstrate a NOTCH–WNT–BMP signal axis as a key crosstalk mechanism between endocardium and myocardium essential for EMT and early heart valve development.

As discussed above, mouse embryos with conditional knockout of myocardial *Bmp2* exhibit decreased expression of *Notch1* in the endocardial cells (58). This observation further supports a crosstalk between myocardial BMP and endocardial NOTCH signaling during EMT and endocardial cushion formation. Interestingly, the expression pattern of *Hey1* and *Hey2*, two well-known targets of the NOTCH signaling, is complementary to *Bmp2* (and *Tbx2*) (96–98). Deletion of *Hey1* and *Hey2* simultaneously results in the expansion of AVC myocardium evidenced by the extended expression of *Bmp2* to the atrial myocardium (97, 98). While *Hey1* and *Hey2* are normally expressed by the atrial and ventricular chamber myocardium, respectively, and absent in the AVC myocardium, their chamber expression can be suppressed by the ectopic expression of *Tbx2* (97, 98). In contrast, overexpression of NICD, *Hey1*, or *Hey2* in the chicken heart inhibits the expression of *Bmp2* in AVC (62). These findings support that this negative feedback loop between the endocardial NOTCH and myocardial BMP signaling defines the regional gene program in the chamber and non-chamber myocardium, which regulates the cell fates of VECs during EMT and early heart valve formation (98, 99).

## Notch Signaling in Aortic Valve Remodeling and Embryonic Pathogenesis Of BAV

After EMT, the cellularized endocardial cushions undergo a complex remodeling process and eventually give rise to the valve leaflets and septa, which divide the heart into four chambers in mammals (Figure 3B). The remodeling process is coordinated by multiple cell populations, including VECs and VICs of different embryonic origins in VECs, NCCs, and SHF. Signals from these cells precisely control the spatiotemporal cell proliferation and apoptosis, as well as dynamic ECM production and organization, which together ensure the proper transformation from the blunted bulky valve primordia into the elongated thin valve leaflets (1, 100). Alterations of these signals can cause congenital heart defects. For example, mutations in the NOTCH pathway genes have been identified to be associated with congenital heart defects. In particular, the *NOTCH1* mutations are among the first genetic variants identified for familial BAV in non-syndromic autosomal-dominant human patients (32). Different *NOTCH1* mutations have been observed in BAV patients by several groups (101–104). Furthermore, mutations in *JAG1* or *NOTCH2* cause Alagille syndrome (AGS), which has defects in multiple organs including the heart valves, and mutations in *JAG1* or *NOTCH1* have also been identified for a subset of tetralogy of Fallot patients (81–83).

Gene expression studies with RNA *in situ* hybridization and immunostaining have found that *Notch1* and *Notch4* are expressed in the endothelial cells, whereas *Notch2* and *Notch3* are present in the smooth muscle cells of the aorta and the pulmonary artery. *Jag1* is broadly expressed in the endothelium, the NCC-derived mesenchyme, and the OFT myocardium (79, 95). Inhibition of NOTCH signaling by overexpressing a dominant-negative version of MAML (MNMAML) specifically in the cardiac NCC lineage using the *Pax3*^Cre^ or *Wnt1*^Cre^ driver in mice causes defects in aortic arch patterning and ventricular septum and pulmonary artery stenosis, which reminisce many OFT defects seen in AGS (79, 95). Analysis of the mutant mouse embryos indicates that loss of NOTCH in the cardiac NCC lineage impairs the differentiation of NCC precursors into smooth muscle cells (79), whereas it has no effect on cell proliferation or migration. In addition, the deletion of *Jag1* or the inhibition of NOTCH in the SHF lineage results in defects in the aortic arch and ventricular septum, as well as thickened semilunar valve leaflets where the valve defect is attributed to the decreased cell apoptosis and disorganized ECM (21). Similar defects are present in the mice with the endothelial specific deletion of *Jag1* (93). Furthermore, genetic interactions between the NOTCH pathway genes and other vascular essential genes are observed in the mouse models of cardiac OFT malformations including BAV. For instance, the study of the compound heterozygous mice for *Notch1* and *Nos3* has suggested that genetic interactions between the NOTCH and endothelial nitric oxide synthase (eNOS) signaling pathways are essential for aortic valve remodeling (105).

Pan-endothelial deletion of NOTCH pathway genes using *Tie1*^Cre^ or *Tie2*^Cre^ as a driver in mice causes severe vascular defects that result in embryonic lethality at the middle gestation (80), excluding the investigation of the functions and mechanisms of NOTCH signaling in regulation of aortic valve remodeling after EMT. In addition, Cre drivers including the endocardial specific Cre *Nfatc1*^Cre^ that mediate gene deletion before EMT disrupt genes in VECs and their daughter VICs, thereby complicating the phenotypic interpretation and molecular signaling identification specific to the post-EMT valve remodeling. To overcome these limitations and specifically study the roles of molecular signals in VECs during aortic valve remodeling, we have developed a new Cre driver termed *Nfatc1*^enCre^, in which the expression of Cre is under the control of an *Nfatc1* gene enhancer (106, 107). Functional validation shows that the *Nfatc1*^enCre^ mice activate the LacZ reporter gene expression specifically in VECs, but not in VICs, beginning around E11.0 (106). We have then generated mouse models with VEC-specific deletion of *Notch1* or *Rbpj*. In contrast to the early lethal phenotype observed in the pan-endothelial *Notch1* or *Rbpj* knockout mice, our VEC-specific *Notch1* mutant mice can survive into adulthood, thereby allowing us to study the role of NOTCH1 signaling in valve remodeling. We have found by echocardiography that the adult VEC *Notch1* conditional knockout mice display increased peak velocity across the aortic valve, indicating a phenotype of aortic valve stenosis (52). Histological and pathological analyses have further indicated that the aortic valves of the mutant mice are thickened, fibrotic, and calcified. In the most severe cases, the cartilage-like structures are present in the leaflets and aortic roots (52). Significantly, some of the mutant mice have developed BAV due to the fusion of the two coronary leaflets (52). These findings demonstrate that the VEC-specific *Notch1* knockout mice recapitulate the common BAV seen in CAVD patients associated with the *NOTCH1* mutations. Thus, the new mouse model offers an excellent opportunity to study the developing mechanism of BAV caused by the *NOTCH1* mutations.

BAV affects ~2% of general populations and is the most common congenital heart malformation (2, 13, 100, 108, 109). It is associated with a high incidence of CAVD and other cardiac complications including aortic dilation, aneurism, and dissection, which can lead to sudden death (8, 9, 110). A BAV may occur isolated or present in a complex syndrome such as Turner, DiGeorge, or Shone syndrome (111, 112). BAV can be caused by the fusion of two of three leaflets or the absence of any one leaflet and the fusion of the right and left leaflet (R–L) is the most common type present in ~70% of all the BAV patients (100). Studies using pig (113), hamster (114–117), and mice models (52, 118) have demonstrated that BAV arises from abnormal aortic valve development. As discussed previously, the *NOTCH1* mutations are associated with the common R–L type of BAV in humans (32). In our mouse studies, we find that the VEC-specific deletion of *Notch1* results in BAV due to the fusion of the left and right coronary leaflet during the early post-EMT remodeling of the aortic valve. At the cellular level, loss of *Notch1* in VEC causes increased proliferation but decreased apoptosis of the VICs (Figure 3B). In contrast, overactivation of NOTCH in VECs results in decreased proliferation and increased apoptosis of VICs, leading to the hypoplastic valves (52). At the molecular level, we show that NOTCH1 expressed in VECs promotes the expression of *Tnfa*, which subsequently induces the apoptosis of VICs. Of note, similar aortic valve remodeling defect has been found by another study using the same Cre driver to delete *Jag1, Notch1*, or *Rbpj*, indicating that the JAG1–NOTCH1 signaling in VECs is required for aortic valve remodeling (78). In this study, loss of NOTCH signaling in VECs leads to increased proliferation of VICs accompanied by decreased expression of *Hbegf*, which is a negative regulator of proliferation through restricting BMP signaling (119). Further molecular studies demonstrate that *Hbegf* is a direct NOTCH target gene in VECs for the proliferation of VICs, as the supplement of soluble HBEGF to the cultured OFT explants from the *Jag1* mutant embryos can rescue the hyperproliferative phenotype (78). These findings support a dual function of the NOTCH1-regulated signaling from VECs in the apoptosis and proliferation of VICs through tumor necrosis factor α and HBEGF, respectively (Figure 3B). These findings are also consistent with the fact that VICs in front of a growing leaflet are highly proliferative, whereas those on the rear side are more likely apoptotic (52). Thus, the NOTCH1 signaling is a critical determinant that controls the balanced proliferation and apoptosis of VICs, which sculpts the endocardial cushions into the thin valve leaflets.

## Notch Signaling in Aortic Valve Homeostasis and Calcification

The aortic valve leaflets, unique tissue microarchitecture with endurant and flexible characteristics essential for a lifelong cyclic function, consist of three layers including fibrosa, spongiosa, and ventricularis (9). The fibrosa layer is located at the aortic side and made up of collagen-rich matrix, VICs, and VECs facing the aortic wall. The spongiosa is the middle layer containing VICs embedded in glycosaminoglycan-rich matrix. The ventricularis layer is rich in elastin fibers and consists of VICs and surface VECs facing the left ventricle (4). VECs on the surface of a leaflet form a barrier that regulate the valve permeability, the adhesion of inflammatory cells, and paracrine or signaling including mechanosensory signals from VECs to VICs (9). VICs are the major cell population in a leaflet, producing ECM and controlling the ECM organization during valve remodeling. In the adult valve, VICs are quiescent under the physiological condition but become activated upon the stimulation by molecular and mechanical signals including the high blood pressure, altered hemodynamics, and inflammatory molecules (9). The activated VICs then undergo transdifferentiation into synthetic myofibroblast and produce many ECM proteins, resulting in pathological ECM remodeling, which makes the valve prone to calcification (5–8).

There is no cure for advanced CAVD patients, except for valve replacement. However, because of high risks of the surgery for the elderly patients and postoperative complications with anticoagulant medicines, the surgical treatment is not suitable for all CAVD patients. Previously considered as a passive consequence of aging (7), CAVD is now recognized as an active disease driven by chronic inflammation (120). Molecular signaling pathways underlying inflammation are thus potential targets for the disease diagnosis and intervention. Signal molecules include eNOS (121), renin–angiotensin system (122–124), NOTCH (89, 90), WNT/β-catenin (122), BMP/TGF-β signaling (122), RANK/RANKL pathway, vitamin K–dependent proteins (125), and microRNAs (9, 126). Here we focus on the NOTCH signaling, whereas the other pathways and molecules are reviewed in recent articles (9, 127).

Since the initial identification of two *NOTCH1* mutations associated with BAV and CAVD by Garg et al. (32), additional *NOTCH1* mutations have been identified in BAV patients by several groups around the world (101, 103, 104). In addition, genome-wide association study has identified several genetic variants in *GATA4/5/6* genes associated with BAV patients (128–130), whereas the targeted disruption of *Gata5* in mice leads to BAV accompanying impaired NOTCH signaling (128, 131, 132). The studies using animal and tissue culture models have further explored the underlying molecular mechanisms. Studies with RNA *in situ* hybridization show that murine *Notch1* mRNA is abundant in VECs and VICs immediately underneath VECs between E11.5 and E13.5 when the intensive valve remodeling is undergoing (32). A similar expression pattern is observed in the adult aortic valves by using the same method. However, antibody staining shows the NOTCH1 protein expression in VECs, but not VICs (52, 78, 89, 92). By using a transgenic mouse line that reports all NOTCH activities via the consensus RBPJ binding sites, we have shown that the NOTCH signaling is highly activated in VECs but absent in VICs during aortic valve development (52). In contrast, in the adult aortic valves, the NOTCH activities are greatly reduced in VECs, whereas some VICs display the NOTCH signals (52). In addition, a reduction of nuclear NOTCH1 activity is seen in the calcified fibrosa when comparing to the non-calcified regions of the diseased valves (90, 133). Together, the dynamic expression pattern of NOTCH1 in the aortic valve indicates potential dual functions in prenatal aortic valve remodeling vs. postnatal homeostasis.

Indeed, the functional studies using mouse models, cultured aortic VICs, and stem cell–derived endothelial cells have collectively revealed the critical roles and mechanisms of NOTCH1 in the regulation of valve calcification. Western diet can induce a 5-fold more calcification in the heterozygous *Notch1* null mice (89). Similarly, after 16 weeks of hyperlipidemic diet feeding, the heterozygous *Rbpj* mice exhibit milder histopathologic features of CAVD including macrophage infiltration, enhanced collagen deposition, pro-osteogenic protein expression, and calcification, but have no obvious hemodynamic changes (91). Endothelial deletion of *Jag1* results in the formation of calcific nodules in the aortic valve of adult mice (93). We have generated and studied a conditional *Notch1* knockout mice model in which *Notch1* was specifically deleted in VECs, and the mutant mice display the typical phenotypes of aortic valve stenosis at a young age of 3 months (52). The mouse CAVD is associated with increased collagen and glycoproteins and calcific nodules. In the most severe case, the cartilage-like structure was seen in the valve leaflet and the annulus (52). Importantly, the mouse CAVD is proceeded with embryonic thickening of the aortic valve leaflets, with a partial penetration of BAV resulting from the fusion of two coronary leaflets resembling the most common form of BAV in humans. In addition, *Nos3*^−/−^; *Notch1*^+/−^ mice develop aortic valve disease, indicating a genetic interaction between *Nos3* and *Notch1* in calcific progression (134, 135). In line with these *in vivo* findings, inhibition of NOTCH in cultured aortic VICs from sheep, rat, or pig promotes calcific nodules formation and osteogenic gene expression.

**Figure 4:**
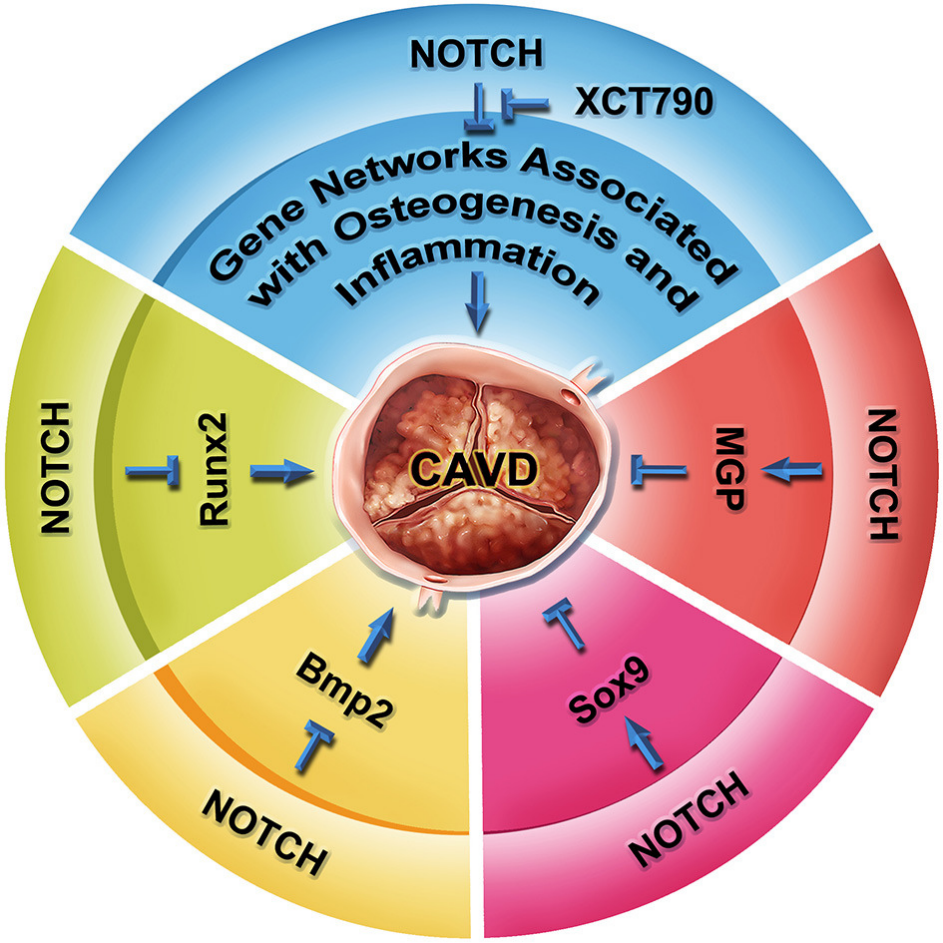
NOTCH signaling in aortic valve homeostasis and CAVD. RUNX2 is a key regulator of osteogenesis, as well as hallmark of CAVD progression. NOTCH represses the transcription of *Runx2* mediated by HEY proteins. NOTCH also represses the expression of *Bmp2* and BMP2-regulated osteoblast-like calcification pathway. Inhibition of NOTCH leads to calcification via a *Sox9*-dependent manner. Conversely, matrix Gla protein MGP suppresses the calcification of soft tissue through antagonizing the BMP signaling. NOTCH positively regulates the transcription of *MGP*. In human iPSC studies, NOTCH1 haploinsufficiency causes gene network change involved in osteogenesis, inflammation, and oxidative stress through an epigenetic mechanism, which can be reversed by a small molecule XCT790. The XCT790 treatment can slow down calcification in the human iPSCs and a mouse model of CAVD.

Studies using primary VICs from multiple species and endothelial cells differentiated from the human induced pluripotent stem cells (hiPSCs) of patients with the *NOTCH1* mutations have begun to uncover the deep molecular mechanisms underlying the CAVD progression associated with the *NOTCH1* haploinsufficiency in humans (Figure 4). The NOTCH1 signaling activates the expression of *Hey1* and *Hey2*, which in turn represses the transcription of *Runx2*, a key transcriptional regulator of osteoblast cell fate (32). *Bmp2* mediates the induction of osteogenic gene expression and calcification by the NOTCH inhibition (89). Genome-wide screening also finds NOTCH1 positively regulates the expression of matrix Gla protein (MGP), which is known to inhibit calcification through sequestering BMPs (136). In cultured aortic VICs, inhibition of the NOTCH signaling results in decreased expression of *Sox9*, and the loss of *Sox9* function is associated with aortic valve calcification (90). Conversely, the supplementation of *Sox9* is able to attenuate the calcification resulting from the NOTCH inhibition. These findings suggest *Sox9* as another downstream mediator of the NOTCH signaling in the regulation of aortic valve calcification. In a coculture system, nitric oxide (NO) produced by endothelial cells inhibits the calcification of VICs, whereas overexpression of *Notch1* can cancel out the calcification of VICs induced by the NO inhibition (134, 137). Recently, Majumdar et al. report that NO mitigates the aortic valve calcification by activating NOTCH signaling through S-nitrosylation of USP9X (137). These findings support interactions between NO and NOTCH1 in aortic valve calcification. In human studies, analysis of the blood samples from 61 patients with aortic stenosis has revealed an association between the *NOTCH1* variants and the OPG/RANKL/RANK gene program (138). In addition, modeling CAVD in endothelial cells differentiated from hiPSCs by introduction of the *NOTCH1* mutations from patients with BAV and aortic valve stenosis has found that the *NOTCH1* haploinsufficiency leads to the alteration of epigenetic architecture, resulting in derepression of latent pro-osteogenic and proinflammatory gene networks (139). Furthermore, a critical role of the telomere shortening in the initiation of calcific program has been reported in the *Notch1*^+/−^ mice (140). More recently, by using hiPSC technology and machine learning, Theodoris et al. have identified a small molecule XCT790, which is able to correct the gene network changes resulting from the *NOTCH1* haploinsufficiency and to prevent the disease progression in a mouse model of CAVD (141). Although multiple observational studies and randomized controlled trials remain needed, XCT790 represents a promising drug for treating CAVD disease in clinical. Together, these current findings reveal a crucial role of NOTCH1 in aortic valve calcification, while the underlying mechanisms remain an area of intensive investigation.

## Conclusions and Perspectives

Heart valve development is a precisely controlled morphogenic process involving cell differentiation, proliferation, apoptosis, and migration, as well as ECM organization. Alteration of molecular signals that regulate this process can cause congenital heart valve defects including BAV, which may lead to CAVD with severe clinical consequences. The structural defects predispose the affected valves to calcification. By using human genetics, animal models, and tissue culture systems, clinicians and basic scientists have worked together and demonstrated the important functions of NOTCH1 in regulation of aortic valve development and CAVD.

The basic and translational research on NOTCH signaling in regulation of aortic valve development and CAVD has led to the identification of a small molecule XCT790 that can halt the disease progression in a mouse model of CAVD. This exemplifies a new avenue for effective drug discovery. However, CAVD is a disease affected by many factors, whereas NOTCH haploinsufficiency contributes to only a small subset of the inheritable cases. Future studies will be needed to broaden the research area to (1) identify additional genetic variants that cause BAV and CAVD using unbiased approaches such as next-generation sequencing, (2) decipher deeper mechanisms underlying disease progression in CAVD patients with or without BAV, and (3) reveal the shared mechanisms among BAV-associated and acquired CAVD that will facilitate the development of a common therapeutics suitable for all patients. It is expected that combination of clinical studies using advanced next-generation sequencing for large patient cohorts and basic researches with hiPSC technology, machine learning, CRISPR-guided gene editing, and single-cell technologies will uncover new BAV and calcific genes, informing pathogenic mechanisms of CAVD and targeted gene therapeutics for the disease.

## Author Contributions

YW and BZ wrote the manuscript. PL, BW, and YF read and critically edited the manuscript. YF drew the figures. All authors contributed to the article and approved the submitted version.

## Conflict of Interest

The authors declare that the research was conducted in the absence of any commercial or financial relationships that could be construed as a potential conflict of interest.
